# γδ T Cells Are Involved in Acute HIV Infection and Associated with AIDS Progression

**DOI:** 10.1371/journal.pone.0106064

**Published:** 2014-09-04

**Authors:** Zhen Li, Wei Li, Ning Li, Yanmei Jiao, Dexi Chen, Lianxian Cui, Yu Hu, Hao Wu, Wei He

**Affiliations:** 1 Department of Immunology, School of Basic Medicine, Peking Union Medical College; National Key Laboratory of Medical Molecular Biology, Institute of Basic Medical Sciences, Chinese Academy of Medical Sciences, Beijing, China; 2 Center for Infectious Diseases, Beijing You'an Hospital, Capital Medical University, Beijing, China; 3 Beijing You'an Hospital, Capital Medical University, Beijing, China; Beth Israel Deaconess Medical Center, Harvard Medical School, United States of America

## Abstract

**Background:**

Early diagnosis is vital to HIV control. γδ T cells play critical roles in viral infections, but their activation in acute HIV infected patients and follow up to 18 months has not been described.

**Methods:**

Changes in γδ T cells, including subsets, function and activation, in treated and untreated acutely HIV-infected patients (n = 79) were compared by cytotoxicity assay and flow cytometry with healthy controls (n = 21) at month 0, 6, 12 and 18.

**Results:**

In acutely HIV-infected patients, Vδ1 cell proportion was elevated (*P* = 0.027) with Vδ2 population reduced (*P* = 0.002). Effector and central memory γδ T cell factions were decreased (*P* = 0.006 and *P* = 0.001, respectively), while proportion of terminal γδ T cells increased (*P* = 0.002). γδ T cell cytotoxicity was compromised over time. Fraction of IL-17-producing cells increased (*P* = 0.008), and IFN-γ-producing cells were unaffected (*P* = 0.115). Elevation of a microbial translocation marker, sCD14, was associated with γδ T cell activation (*P* = 0.001), which increased in a time-dependent manner, correlating with CD4/CD8 T cell activation set-points and CD4 counts. Antiretroviral therapy did not affect these changes.

**Conclusions:**

γδ T cell subpopulation and functions change significantly in acute HIV infection and over time. Early γδ T cell activation was associated with CD4/CD8 T cell activation set-points, which predict AIDS progression. Therefore, γδ T cell activation represents a potential surrogate marker of AIDS progression.

## Introduction

Human γδ T cells are distinct from αβ T cells, and generally lack CD4 or CD8 antigen expression [1]. These cells represent approximately 3–4% of peripheral T cells in the blood, and are composed predominantly of two subsets, depending on expression of the Vδ1 or Vδ2 gene [2]–[5].Vδ1 T cells respond to antigens from bacterial pathogens [6]. Vδ2 T cells are believed to be activated by several chemical compounds, some of which are produced by activated host cells in response to pathogenic infections [7]. Vδ2 T cells can be further divided into four subtypes: naïve (Tnaïve), central memory (TCM), effector memory (TEM) and terminally differentiated (TEMRA), based on differential surface expression of CD45RA and CD27 [8], [9]. Specific Tnaïve and TCM cells are located in the lymph nodes and possess distinct functions [10]–[12]. TEM and TEMRA are located preferentially at inflammatory sites and perform effector functions [10].

Functionally, γδ T cells produce interleukin 17 (IL-17), interferon gamma (IFN-γ), and other secreted protein factors after activation [13], [14]. IL-17 is thought to be important for maintaining intestinal mucosal integrity and controlling microbial translocation [15]. IFN-γ levels are elevated in many proinflammatory events, and microbial translocation [16]. Furthermore, γδ T cells are involved in the recruitment of neutrophils in bacterial infections [17]. Reports indicate that, the cytotoxic activity of γδ T lymphocytes against human neuroblastoma cells, Burkitt's lymphoma cells and various cancer cell lines (human colon carcinoma, erytholeukemia, human neuroepithelioma and renal adenocarcinoma) is augmented upon activation [18], [19].

It has been reported that, in all stages of HIV infections, gut functions are compromised, leading to enteropathy, which is commonly observed in HIV-infected patients. The permeability of the mucosal barrier is significantly elevated [20], [21], which may result in translocation of microbial products into the systemic circulation, leading to immune stimulation. A number of studies have described the use of soluble CD14 (sCD14) as a marker of microbial translocation, and even as a predictive marker of disease progression in HIV infection [22]–[24].

The γδ T cell responses to HIV infection are complicated and not well-elucidated [25], [26]. Previous reports have indicated increased levels of Vδ1 cells and decreased levels and function of Vδ2 cells in chronically HIV-infected patients [26], [27]. Furthermore, changes in the Vδ2 T cell population correlated positively with CD4 T cell counts and negatively with viral loads [26].

The therapeutic efficacy of antiretroviral therapy (ART) on Vδ2 T cell recovery in HIV patients is controversial and inconclusive. A report in 2004 indicated partial recovery of the Vδ2 T cell population following long-term HIV-suppressive therapy [28]. However, a subsequent study demonstrated that ART failed to restore the Vδ2 repertoire in HIV-infected men [29].

In this study, we aimed to characterize the changes in γδ T cells, including subsets, function and activation status, in acutely HIV-infected patients who were followed from acute infection up to 18 months, and to define their possible correlation with other serum parameters, microbial translocation and AIDS progression.

## Methods

### Study subjects

The protocol for this study was approved by the Beijing You'an Hospital Ethics Committee and the Guidelines for Human Experimentation (PR. China) were followed throughout. Upon admission, all patients provided written informed consent for their information and clinical samples (blood and plasma) to be stored and used for research.

Seventy-nine homosexual men with acute HIV infection were enrolled in a prospective study (co-inclusion in the Beijing PRIMO cohort) conducted in Beijing You'an Hospital, Beijing, PR. China. Acute HIV infection was defined by a negative enzyme-linked immunosorbent assay (ELISA), and at least one of the following criteria: less than three bands on HIV Western blot (HIV BLOT2.2, 11039, Diagnostic Biotechnology Pte, Singapore), a positive p24 test or detectable plasma HIV-RNA. The estimated date of infection was calculated as follows: 2 weeks before onset of symptoms or 4 weeks before the first positive Western blot. All 79 enrolled HIV-infected patients were diagnosed early during the acute stage of HIV infection (median of estimated time post-infection: 41 days) and transmitted by homosexual activity. At baseline (day 0 of enrollment), all patients were treatment-naïve. During the follow-up times, some of the patients (11/79) started combination antiretroviral treatment (ART), based on CD4 cell counts (e.g. <350/µL according to WHO recommendations) with or without clinical symptoms, and the consensus of both physicians and patients. Patients who were treated during the study received a combination of AZT, 3TC, and EFV or LPV/r. The courses of their treatment were not influenced by the authors for the purpose of this study. Most of them (68/79) were still untreated [CD4 counts: >350/µL (n  =  48), <350/µL (n = 20)]. Twenty-one male homosexual healthy subjects were included as controls (HC). The ages of all groups were matched.

Blood samples were collected from patients at baseline (day 0 of enrollment), month 6, month 12 and month 18. Plasma samples were also collected from all healthy volunteers. All tests were performed at baseline. In addition, CD38/HLA-DR antigen expression and the cytotoxicity activity of γδ T cells were determined at baseline, month 6, month 12 and month 18.

### Cell culture

Peripheral blood mononuclear cells (PBMCs) were isolated by Ficoll-Hypaque (TBD, Tianjin, China) centrifugation. The expansion of γδ T cells was performed as previously described [30]. Briefly, 24-well plates were coated with 500 µL purified anti-γδTCR antibody (IMMU510, 1 mg/mL; Immunotech, Beckman Coulter, Fullerton, CA, USA) at 37°C for 2 h. PBMCs were then added to the antibody-coated wells and cultured in RPMI 1640 medium supplemented with 10% FCS and 200 IU/mL recombinant human IL-2 (Beijing Read United Cross Pharmaceutical, Beijing, China). The culture medium was replaced every other day. The trypan blue exclusion method was employed to assess cell viability.

### Antibodies and flow cytometry

Antibodies (Abs) used in this study were as follows: mouse phycoerythin (PE)-conjugated anti-human CD38 (HIT2) Ab, mouse allophycocyanin (APC)-conjugated anti-human HLA-DR (L243) Ab, mouse phycoerythin-cyanin7(PE-cy7)-conjugated anti-human CD3 (HIT3a) Ab, mouse peridinin-chlorophyll-protein complex cyanine 5.5 (PerCP-cy5.5) conjugated anti-human CD27 (O323) Ab, mouse APC-conjugated anti-human CD45RA (HI100) Ab were purchased from Biolegend (San Diego, CA, USA). Mouse Fluorescein isothiocyanate (FITC)-conjugated anti-human pan TCRγδ Ab, mouse PE-conjugated anti-human pan TCRγδ (IMMU510) Ab and mouse FITC-conjugated anti-human pan Vδ_2_TCR (IMMU389) Ab were purchased from Immunotech (Beckman Coulter, Fullerton, CA, USA). Mouse FITC-conjugated anti-human pan Vδ_1_TCR (TS8.2) Ab was purchased from Pierce (USA). Mouse PE-conjugated anti-human IL-17A (SCPL1362) Ab and mouse PE-conjugated anti-human IFN-γ (B27) Ab were purchased from BD Pharmingen (San Diego, CA, USA). The isotype control antibodies were purchased from the corresponding company, respectively.

Cells were stained with appropriate target Abs and isotype Abs using conventional surface- and/or intracellular staining methods. When both surface and intracellular staining was required, cells were first fixed and permeabilized using BD Cytofix/Cytoperm Fixation and Permeabilization Solution (BD Pharmingen), followed by staining for intracellular proteins. Cells were then washed extensively with PBS to remove excess Ab, stained for extracellular targets, and fixed with 2% formaldehyde. Fluorescence was evaluated with a FACSCalibur flow cytometer, and data were analyzed using FlowJo software (Tree Star, Ashland, OR, USA).

### Cytotoxicity

The CytoTox 96 Non-Radioactive Cytotoxicity Assay (Promega, Madison, WI, USA), based on the colorimetric detection of released lactate dehydrogenase (LDH), was used according to the manufacturer's instructions. Tumor cells used as target cells were seeded into a 96-round well plate (5×10^4^ cells/well). Expanded γδ T cells (effector cells) were added directly to individual wells at different effector/target (E/T) ratios. The plate was incubated at 37°C for 4 h before supernatants were collected and LDH activity was determined. Controls for spontaneous LDH release from effector and target cells, the target maximum release, as well as the culture medium background were assayed simultaneously. The cytotoxicity was calculated as follows: %Cytotoxicity  =  (Experimental-Effector spontaneous-Target spontaneous)/(Target maximum-Target spontaneous) ×100%.

### Quantification of plasma lipopolysaccharide (LPS), LPS-binding protein (LBP) and soluble CD14 (sCD14)

Commercially available ELISA kits were used in this study following manufacturers' instructions for measuring plasma concentrations of LBP (Cell Sciences, MA, USA) and sCD14 (soluble CD14) (R&D Systems, MN, USA). Plasma LPS levels were quantified using a *Limulus amebocyte* lysate (LAL) assay (Hycult Biotech, UDEN, Netherlands).

### Statistical analysis

Non-parametric tests were used to avoid the impact of potential outlier values in a small study. Comparisons between groups were performed using the Mann-Whitney test. The Wilcox on matched pairs test was used to estimate the changes in the different variables throughout the follow-up. The Spearman's non-parametric correlation was used to estimate the association of two continuous variables of interest. *P*-values below 0.05 were considered statistically significant. Data of patients on ART were compared only with data of untreated patients with CD4 counts lower than 350/µL.

## Results

### Changes in γδ T cell subpopulations in acutely HIV-infected patients

**Figure 1:**
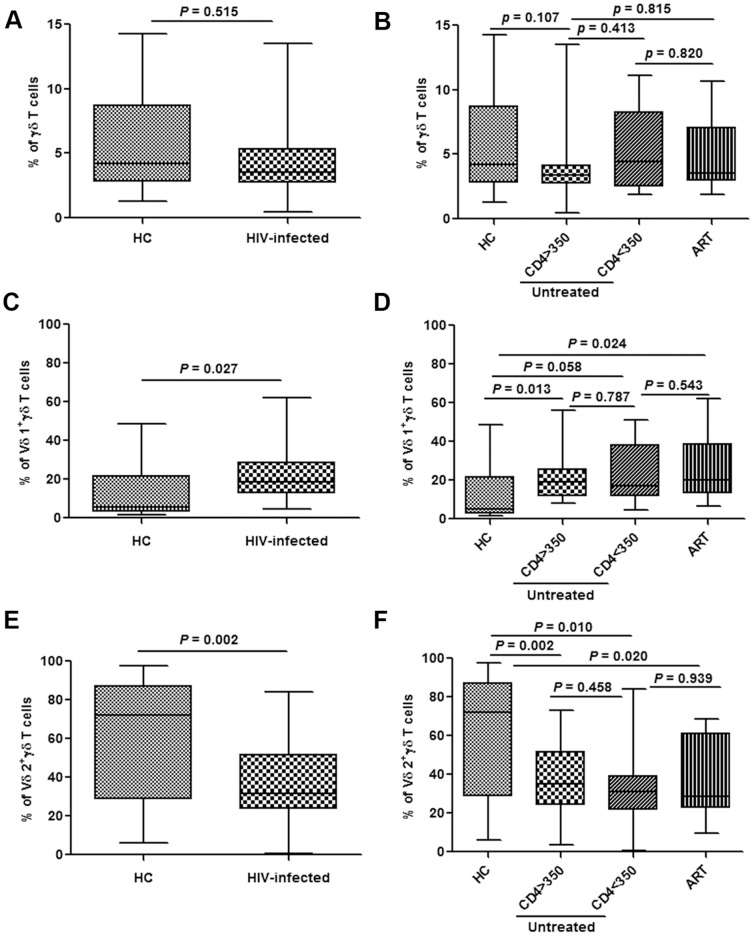
Changes in different subpopulations of γδ T cells in acutely HIV-infected patients. Frequencies of total γδ T cells, Vδ1 γδ T and Vδ2 γδ T cells were analyzed in healthy controls (HC) and acutely HIV-infected subjects at baseline (**A, C, E**). The patients were then subdivided based on the administration of antiretroviral therapy (ART) and CD4 levels (< or >than 350/µL). The frequencies were re-analyzed and compared with HC (**B, D, F**).

Compared with healthy subjects, there was no marked difference in the fraction of γδ T cells in acutely HIV-infected patients (**Fig. 1A**). Nor were there any differences between patients with different CD4 counts, or those who did or did not receive ART (**Fig. 1B**). To characterize the changes in γδ T cells subpopulations in acutely HIV-infected patients, we first analyzed changes in the Vδ1 and Vδ2 subtypes. The proportion of Vδ1 cells among γδ T cells was elevated (*P* = 0.027), while the Vδ2 population was significantly reduced (*P* = 0.002) (**Fig. 1C and 1E**). However, there were no significant differences in both the proportions of Vδ1 and Vδ2 cells between patients with different CD4 counts (both *P*>0.05). Furthermore, Initiation of ART failed to bring about Vδ2 subtype recover, and had no effect on the Vδ1 population (**Fig. 1D and 1F**).

**Figure 2:**
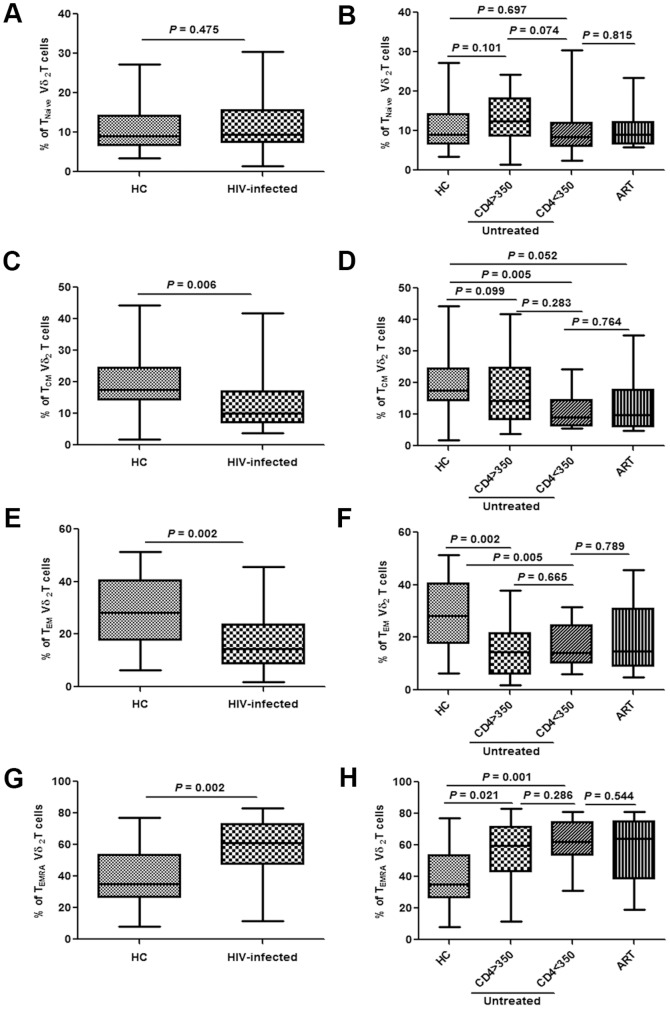
Changes in subpopulations of Vδ2γδ T cells in acutely HIV-infected patients. Frequencies of Tnaïve, TCM, TEM and TEMRA cells were analyzed in healthy controls (HC) and acutely HIV-infected subjects at baseline (**A, C, E, G**). The patients were then subdivided based on the administration of antiretroviral therapy (ART) and CD4 levels (< or >than 350/µL). The frequencies were re-analyzed and compared with HC (**B, D, F, H**).

Changes in the levels of Vδ2 γδ T subgroups in acutely HIV-infected patients were investigated by analysis of the expression of surface CD27 and CD45RA antigens [8], [9]. There was no difference in the proportion of naïve Vδ2γδT cells (CD27+/CD45RA+) observed (*P* = 0.475), on which CD4 levels and ART showed no impact (**Fig. 2A and 2B**). The fractions of the TEM (effector memory Vδ2 γδ T cells, CD27−/CD45RA−) and TCM (central memory Vδ2 γδ T cells, CD27+/CD45RA−) populations were significantly decreased in acutely HIV-infected patients (*P* = 0.002 and *P* = 0.006, respectively), while the proportion of TEMRA (terminal Vδ2 γδ T cells, CD27−/CD45RA+) was increased (*P* = 0.002) (**Fig. 2C, 2E, and 2G**). CD4 levels and the initiation of ART showed no correlation with these changes (**Fig. 2D, 2F, and 2H**).

### Compromised γδ T cell functions in acutely HIV-infected patients

**Figure 3:**
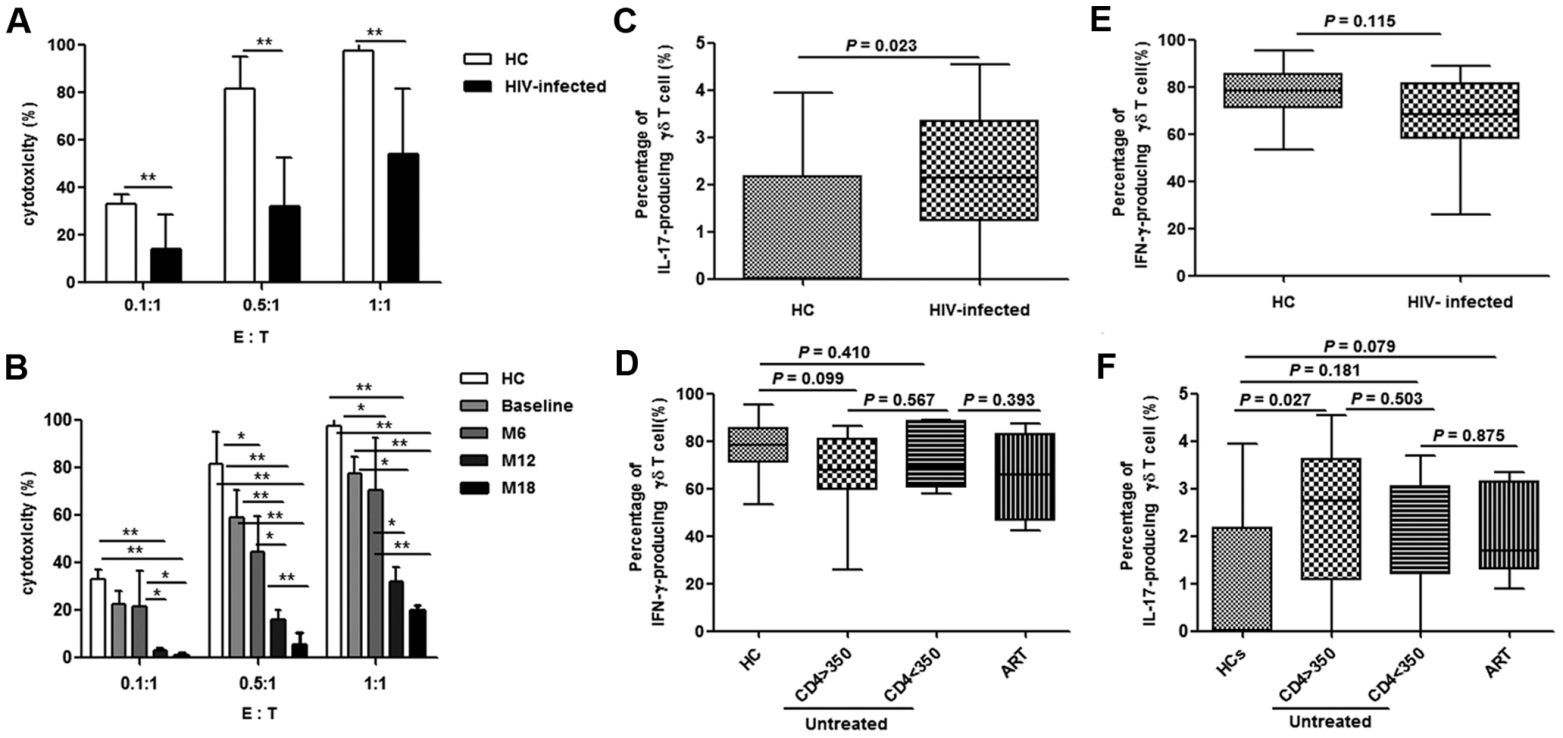
Altered functions of γδ T cells in acute HIV infection. Cytotoxicity (%) was assessed at different effector (E, γδ T cells) to target (T, Daudi cells) ratios (0.1∶1, 0.5µ1 and 1∶1) at baseline (**A**). Subsequently, longitudinal follow-up analysis of cytotoxicity was conducted at different time-points [baseline, month 6 (M6), month 12 (M12) and month 18 (M18)] (**B**). Frequencies of IFN-γ- and IL-17-producing γδ T cells were analyzed in healthy controls (HC) and acutely HIV-infected subjects at baseline (**C, E**). The patients were then subdivided based on the administration of antiretroviral therapy (ART) and CD4 levels (< or >than 350/µL). The frequencies were re-analyzed and compared with HC (**D, F**). *: *P*<0.05, **: *P*<0.01.

The cytotoxic activity of γδ T cells in the acute stage of HIV infection was analyzed at baseline, month 6, 12 and 18. Compared with data from healthy controls, γδ T cell cytotoxicity was gradually compromised in acutely HIV-infected patients over time (**Fig. 3A and 3B**). Furthermore, the fraction of IL-17-producing γδ T cells was elevated (*P* = 0.023), while the fraction of IFN-γ-producing γδ T cells was unchanged (*P* = 0.115) (**Fig. 3C and 3E**). These changes were not affected by CD4 counts or the initiation of ART (**Fig. 3D and 3F**).

### Activation of γδ T cells

**Figure 4:**
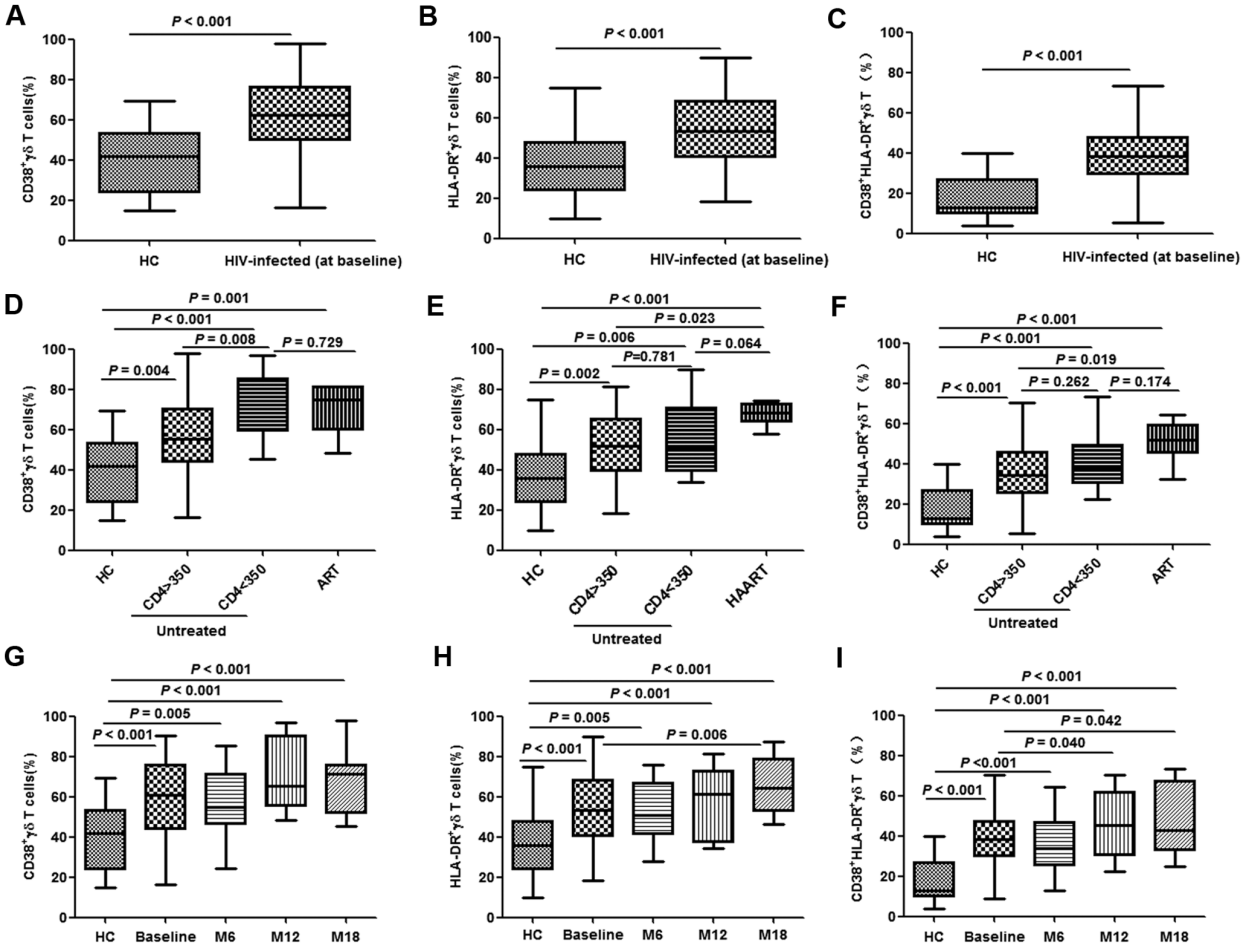
Activation of γδ T cells in acute HIV infection. Activation of γδ T cells was determined by analysis of CD38 and HLA-DR expression. Frequencies of CD38+, HLA-DR+ and CD38+/HLA-DR+ γδ T cells were tested in healthy controls (HC) and acutely HIV-infected subjects at baseline (**A–C**). The patients were then subdivided based on the administration of antiretroviral therapy (ART) and CD4 levels (< or >than 350/µL). The frequencies were re-analyzed and compared with HC (**D–F**). Longitudinal follow-up of γδ T cell activation in patients with acute HIV infection was conducted at baseline, month 6 (M6), month 12 (M12) and month 18 (M18). Frequencies of CD38+, HLA-DR+ and CD38+/HLA-DR+ γδ T cells were tested in healthy controls (HC) and acutely HIV-infected subjects at baseline (**G–I**).

CD38 and HLA-DR are used as surrogate markers of γδ T cell activation [31], [32]. Compared with healthy controls, the proportions of CD38+ or HLA-DR+ γδ T cells (single-positive and double-positive) were significantly elevated in acutely HIV-infected patients (all *P*<0.001) (**Fig. 4A–C**). For patients with a CD4 count of less than 350/µL, the fraction of CD38+ γδ T cells was higher than for those with a CD4 count of greater than 350/µL(*P* = 0.008) (**Fig. 4D**); however, this CD4-dependent change was not evident in HLA-DR+ or double-positive cells (*P* = 0.781 and 0.262, respectively) (**Fig. 4E and 4F**). No significant differences were observed among patients on ART compared with untreated patients with a CD4 count of less than 350/µL (**Fig. 4D–F**).

To further characterize the activation of γδ T cells, CD38 and HLA-DR expression was determined at different time-points (baseline and months 6, 12, and 18). Steady increases in the fractions of all three groups of γδ T cells (CD38+, HLA-DR+ and CD38+/HLA-DR+) were observed over time, except for CD38+ cells, which showed a marginal reduction at month 6 (**Fig. 4G–I**).No correlation was detected between γδ T cell activation and baseline viral load (data not shown).

### Correlations between early γδ T cell activation and CD4/CD8 T cell activation set-point

**Figure 5:**
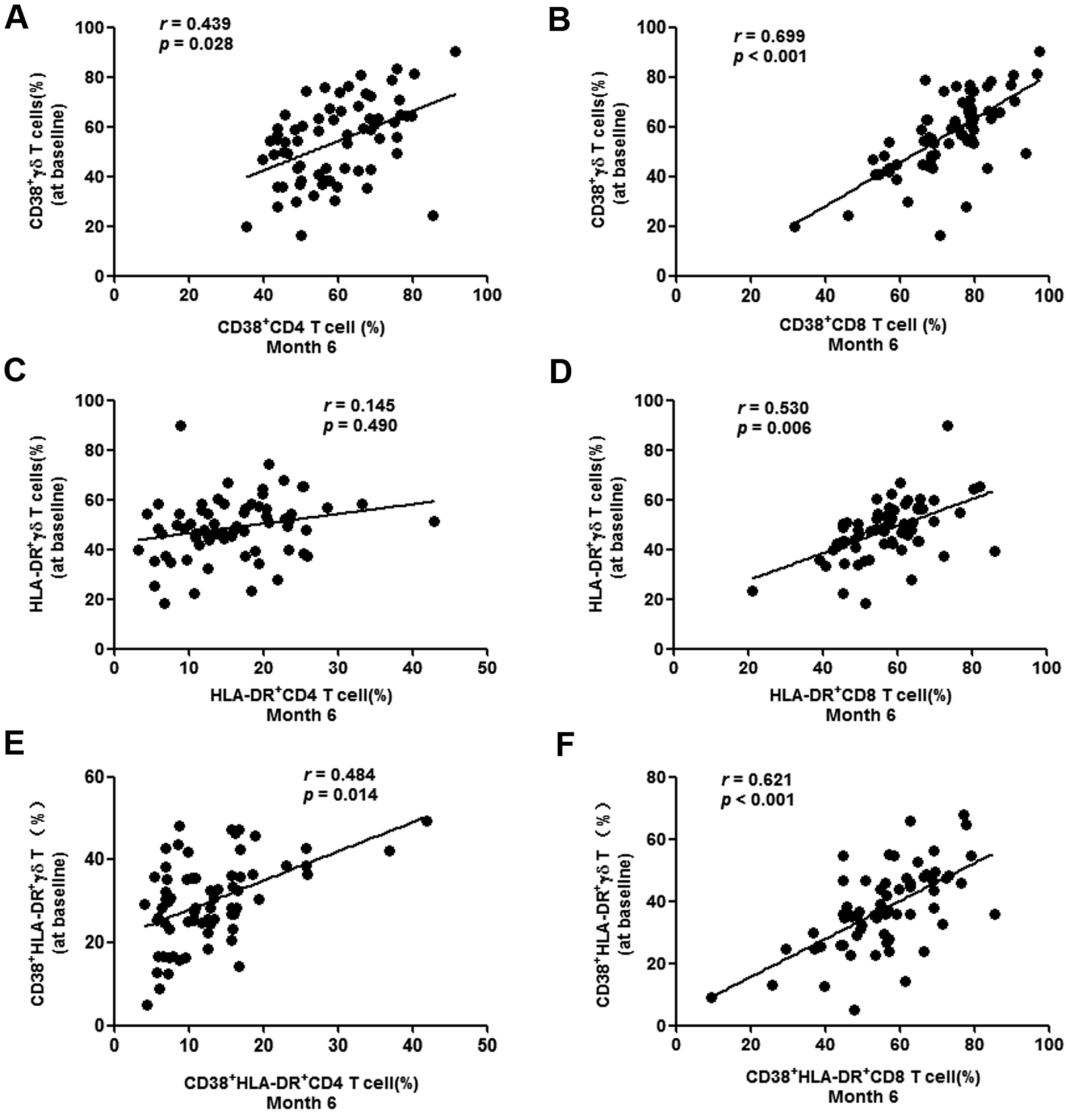
Correlation between early γδ T cell activation and CD4/CD8 T cell activation set-point. Correlation analysis of the proportion of CD38+/CD4+ T cells (**A**) or CD38+/CD8+ T cells (**B**) at month 6 after baseline and CD38+ γδ T cells frequency at baseline was performed by Spearman's rank correlation, where coefficients ‘r’ and corresponding *P*-values are indicated on each panel. Similar correlation analysis of the proportion of HLA-DR+ (**C, D**) and CD38+/HLA-DR+ cells (**E, F**) and CD4+ or CD8+ T cells at month 6 and γδ T cells at baseline was performed.

Activation of CD4/CD8 cells was determined on the basis of CD38 and HLA-DR surface marker expression at month 6 after baseline, when the T cell activation set-point of most patients should have been established [33]. Correlation analysis was performed by comparing CD4/CD8 activation at month 6 and γδ T cell activation at baseline. CD4 and CD8 T cell activation set-points were both positively correlated to γδ T cell activation (**Fig. 5A–B and 5E–F**). If HLA-DR alone was used, the CD8 T cell set-point was correlated (*P* = 0.006), but not the CD4 T cell activation set-point (*P* = 0.490) (**Fig. 5C and 5D**). These data demonstrated that early activation of γδ T cells was positively correlated withCD4 and CD8 T cell activation set-points.

### Microbial translocation versus γδ T cells

**Figure 6:**
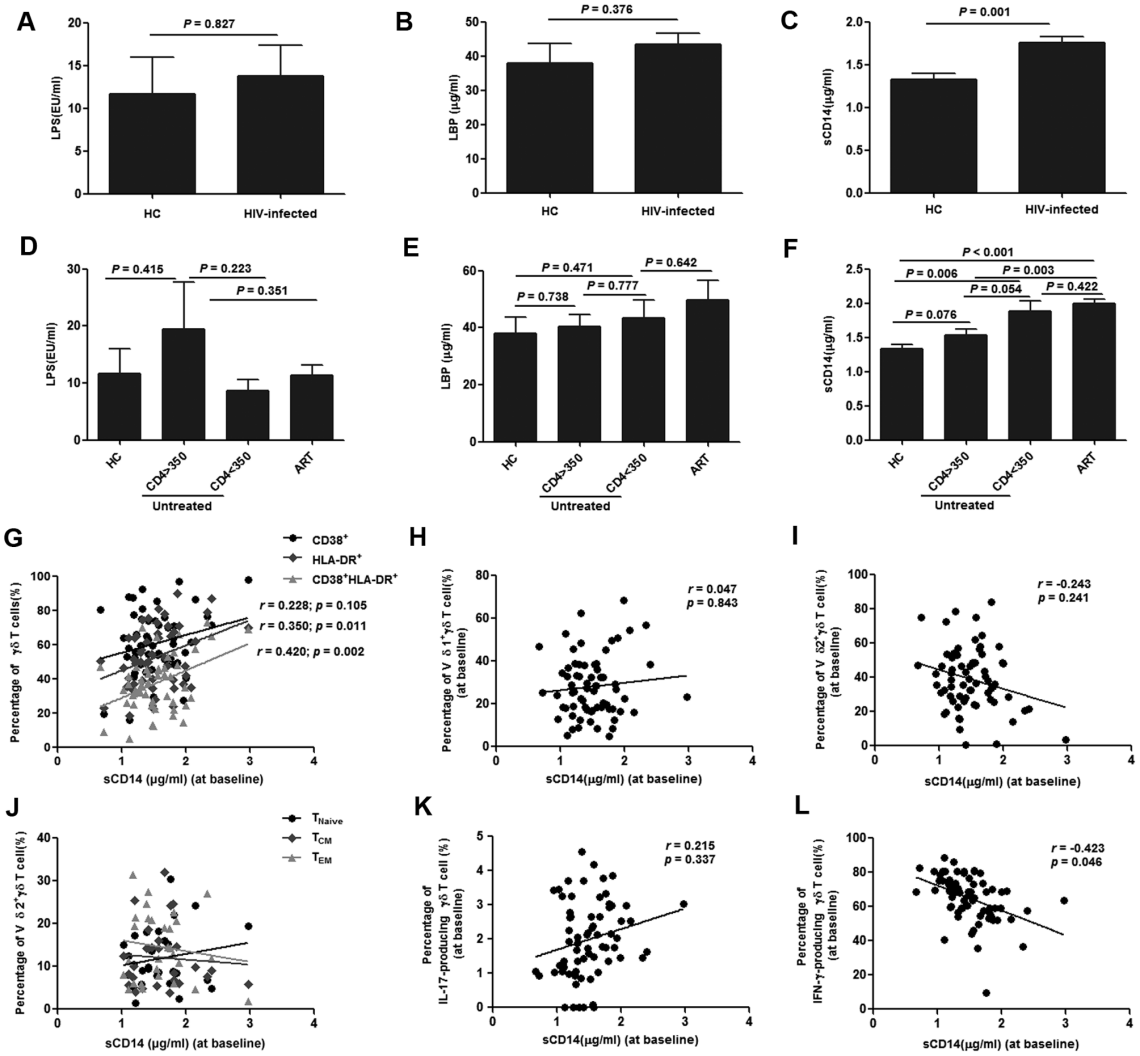
Correlation between microbial translocation and γδ T cells. Levels of LPS, LBP and sCD14 were analyzed in healthy controls (HC) and acutely HIV-infected subjects at baseline (**A–C**). The patients were then subdivided based on the administration of antiretroviral therapy (ART) and CD4 levels (< or >than 350/µL). The levels of LPS, LBP and sCD14 were re-analyzed and compared with HC (**D–F**). Correlation analysis between the proportion of activated γδ T cells determined by CD38+, HLA-DR+ or CD38+/HLA-DR and sCD14 levels at baseline was performed by Spearman's rank correlation, where coefficients ‘r’ and corresponding *P*-values are indicated on panel G. Similar correlation analysis was performed between sCD14 levels and different subsets of γδ T cells [Vδ1 (**H**) and Vδ2 (**I**)], subgroups of Vδ2 cells (Tnaïve, TCM and TEM) (**J**), IL-17-producing γδ T cells (**K**) or IFN-γ-producing γδ T cells (**L**).

Microbial translocation in acute HIV infection was determined by analysis of the serum levels of LPS (lipopolysaccharides), LBP (LPS-binding protein) and sCD14 (soluble CD14). Only sCD14 levels were found to be elevated in acutely HIV-infected patients compared with those of healthy subjects (*P* = 0.001) (**Fig. 6A–C**). The levels of LPS and LBP did not differ between ART-treated and untreated patients, or between patients with different CD4 count levels (**Fig. 6D–E**). However, the sCD14 level in ART-treated patients was significant elevated compared with untreated patients with CD4>350 cells/µl (*P* = 0.003). Correlation analysis also showed no association between sCD14 levels and the total γδ T cell population (*P* = 0.062) (data not shown); however, further investigation demonstrated positive correlations between sCD14 levels and different γδ T subgroups. Statistical analysis indicated that sCD14 levels were positively correlated withCD38+/HLA-DR+ (*P* = 0.002) and HLA-DR+ (*P* = 0.011), but not with CD38+ (*P* = 0.105) (**Fig. 6G**). Fractions of Tnaïve, TCM and TEM were not correlated with sCD14 levels (all *P*>0.05) (**Fig. 6J**). Proportions of Vδ1, Vδ2 and IL-17 levels were not associated with sCD14 levels (all *P*>0.05) (**Fig. 6H, I, K**).The fraction of IFN-γ-producing γδ T cells was negatively correlated with sCD14 levels (*P* = 0.046) (**Fig. 6L**). Furthermore, sCD14 was associated with LPS levels (*P* = 0.034) (data not shown).

## Discussion

There is an increasing body of evidence indicating that γδ T cells play critical roles in HIV infections [25]. In this study conducted in a male homosexual population, we show that the proportion of Vδ1 cells among γδ T cells was elevated in acutely HIV-infected patients, while the Vδ2 population was significantly reduced. Among theVδ2γδT cells, the fractions of TCM and TEM cells were significantly decreased, while the proportion of TEMRA was increased. In term of γδ T cell functions, cytotoxicity was compromised significantly in acutely HIV-infected patients over time. The fraction of IL-17-producing cells was elevated, but not that of the IFN-γ-producing cells. Activation of γδ T cells increased over time and correlated withCD4 counts and CD4/CD8 T cell activation set-points. In acute HIV infection, levels of the microbial translocation marker, sCD14, were significantly elevated, regardless ofCD4 counts. However, sCD14 levels exhibited a positive association withCD38/HLA-DR and HLA-DR expression and a negative association with the fraction of IFN-γ-producing γδ T cells. Initiation of ART had no impact on any of these changes.

It has been shown that the Vδ2 population is depleted in chronically HIV-infected patients [25]. Our data indicated that this is an early event, occurring before the remarkable reduction in the total γδ T cell count, since baseline data showed no significant change in the fraction of γδ T cells among the total T cell population (**Fig. 1A, 1C and 1E**). Our results also demonstrated that there were no significant changes in the proportion of Tnaïve cells in the Vδ2 cell population. The fractions of TEM and TCM cells were decreased, while the TEMRA fraction was elevated, which indicated increased aging of Vδ2 cells in acute HIV infection (**Fig. 2**).

Activated γδ T cells are the subtype of this population of cells that function in response to HIV infections, and therefore, were the primary target cell population of this study. Our data showed a gradual increase in γδ T cell activation, along with compromised γδ T cell cytotoxicity over time, in addition to an elevation in the fraction of IL-17-producing γδ T cells in acute HIV infection (**Fig. 3** **,** **4** **,** **5**).These observations indicated that early initiation of ART failed to bring about recovery of the γδ T cell populations and their functions, which is consistent with a previous report [29], although additional studies are required to confirm this. More importantly, we found a positive correlation between baseline γδ T cell activation and CD4/CD8 T cell activation set-points (**Fig. 5**), which were used to predict subsequent CD4 T cell loss, disease progression and outcome [32], [34]. It can therefore be speculated that activation of γδ cells represents a surrogate marker for disease progression and outcome.

Our results indicated potential associations between γδ T cell activation and levels of sCD14, a monocyte activation marker, which is also commonly used as an indicator of microbial translocation (**Fig. 6**). The level of sCD14 is used to predict mortality and disease progression in chronic HIV patients [24], [33], in addition to other disorders, such as different cancers and hemodialysis [35], [36]. Microbial translocation results in the release of bacterial products, such as LPS and LBP, which in turn activate monocytes in various clinical situations [37], [38]. LPS and LBP levels have been shown to be increased in chronic HIV infection, but not in the acute stage. Furthermore, it has been reported that microbial translocation is not observed in primary HIV infection, although sCD14 levels are elevated [33], [39]. Therefore, even though sCD14 levels were found to be elevated in acute HIV infection in our study, an association of γδ T cell activation with microbial translocation remains to be established.

No differences were observed between patients on early ART and untreated patients in any aspect investigated in this study. Although there are reports indicating that ART does not bring about recovery of the γδ T cell depletion, this conclusion is controversial. Our study was conducted in only 11 patients on ART, and two treatment plans including two different protease inhibitors; therefore, a larger study with a more stringent design is required to confirm this conclusion.

In conclusion, in acute HIV infections, a significant shift in the proportions and functions of the various γδ T cell subgroups was observed. Elevation of the microbial translocation marker, sCD14, was associated with γδ T cell activation. More importantly, early γδ T cell activation was associated with CD4 and CD8 T cell activation set-points, which predict AIDS progression. Therefore, γδ T cell activation represents a potential surrogate marker of AIDS progression. This conclusion requires confirmation in larger prospective cohort studies.
